# Genetically Engineered Mouse Models of Gliomas: Technological Developments for Translational Discoveries

**DOI:** 10.3390/cancers11091335

**Published:** 2019-09-09

**Authors:** Imran Noorani

**Affiliations:** Department of Academic Neurosurgery, Addenbrooke’s Hospital, University of Cambridge, Cambridge CB2 0QQ, UK; imran.noorani@cantab.net

**Keywords:** cancer, glioma, mouse model, CRISPR, piggyBac, transposon

## Abstract

The most common brain tumours, gliomas, have significant morbidity. Detailed biological and genetic understanding of these tumours is needed in order to devise effective, rational therapies. In an era generating unprecedented quantities of genomic sequencing data from human cancers, complementary methods of deciphering the underlying functional cancer genes and mechanisms are becoming even more important. Genetically engineered mouse models of gliomas have provided a platform for investigating the molecular underpinning of this complex disease, and new tools for such models are emerging that are enabling us to answer the most important questions in the field. Here, I discuss improvements to genome engineering technologies that have led to more faithful mouse models resembling human gliomas, including new cre/LoxP transgenic lines that allow more accurate cell targeting of genetic recombination, *Sleeping Beauty* and *piggyBac* transposons for the integration of transgenes and genetic screens, and CRISPR-cas9 for generating genetic knockout and functional screens. Applications of these technologies are providing novel insights into the functional genetic drivers of gliomagenesis, how these genes cooperate with one another, and the potential cells-of-origin of gliomas, knowledge of which is critical to the development of targeted treatments for patients in the clinic.

## 1. Introduction 

Gliomas are the most common intrinsic brain tumours, causing considerable morbidity and mortality. Given that the prognosis of malignant gliomas has not substantially improved in the last few decades, strong efforts are being made into understanding the genetics and biology of this disease in order to design targeted therapies against oncogenic pathways. Although whole-genome sequencing studies of human tumours have provided some insight into the genetic make-up of gliomas in the last few years, disentangling functional driver genes from other ‘passenger’ mutations in these tumours requires complementary studies. Genetically engineered mouse models (GEMMs) of gliomas are powerful experimental tools that can provide new insights into their genetic drivers and molecular mechanisms, in addition to providing resources for preclinical drug testing. Compared with in vitro systems and xenografts in immune-deficient mice, GEMMs in immune-competent mice have the major advantages of being able to model interactions between tumour cells and the microenvironment including the vasculature and immune cells. Such interactions are known to have a critical role in gliomagenesis [1,2,3]. A systematic review of mouse models of gliomas has previously been published [4], and other glioma models have been reviewed [5]. Recent advances in genomic engineering technologies have enabled considerable improvements in glioma GEMMs to be made, and these will be discussed in this Review with prominent examples to highlight their utility for exploring functional genetic drivers. 

Diffuse gliomas may be classified into low- and high-grade gliomas, and include oligodendrogliomas, astrocytomas, and glioblastomas. Low-grade gliomas (LGGs, World Health Organisation, grade II) are a heterogeneous group of intrinsic brain tumours with a tendency for progression to higher-grade tumours. These tumours contain cells with similar histological appearances as glial cells, including astrocytes and oligodendrocytes. LGGs constitute 15% of all adult brain tumours, and they most commonly present with seizures (in 80% of cases) [6]. A model for the natural history of gliomas posits four phases: 1) the occult stage, in which tumour-initiating cells proliferate but there is no detectable tumour on magnetic resonance imaging (MRI); 2) the clinically silent stage, in which tumour mass becomes apparent on MRI but the patient remains clinically asymptomatic (this is termed an incidental glioma) [7]; 3) the symptomatic stage, in which the tumour elicits symptoms or signs such as seizures or weakness; and 4) malignant transformation, in which the LGG switches to a more biologically aggressive high grade glioma [8,9]. Upon malignant transformation, the tumour is termed a secondary glioblastoma. 

Glioblastoma (GBM), or high-grade glioma (HGG), is the most common malignant intrinsic brain tumour, and characteristically invades surrounding brain aggressively, making complete surgical resection of all cancerous cells impossible. The disease tends to affect middle-aged to elderly people and can either arise de novo (primary glioblastoma) or by transformation from an LGG (secondary glioblastoma). Its prognosis is therefore poor, with a median survival of only 14 months despite maximal therapy with surgery and chemo- or radiotherapy [10]. These survival times have not substantially improved over the last few decades despite improvements in treatment. Hence, deeper understanding of the biology of this cancer is needed before significant advances in treatment outcomes can be achieved. 

Temozolomide, a DNA alkylating agent, is the mainstay of chemotherapy for GBM and has been shown in randomised controlled trials to improve prognosis by around two months when given with radiotherapy [10,11]. Given the limited impact this has on survival, there is a strong need for more molecularly targeted treatments for this cancer that will improve prognosis further. A recent example of such a therapy that has entered clinical practice for recurrent GBM is bevacizumab (Avastin): a monoclonal antibody targeting vascular endothelial growth factor (VEGF) that aims to block tumour angiogenesis. Although bevacizumab slows glioma growth, randomised controlled trials have failed to demonstrate it improves overall survival in GBM; however, the evidence shows a benefit in progression-free survival of around 2–3 months in patients with newly diagnosed GBM [12]. Nevertheless, all patients succumb to the disease after developing resistance to treatment. Surgery, although beneficial, is not curative because glioma cells tend to invade well beyond the macroscopically-visible margins of resection, and such cells (possibly glioma stem cells) trigger disease recurrence [13]. A number of genes are implicated in resistance to temozolomide, most are involved in DNA repair, for example *MGMT*, *MSH2*, and *MSH6* [14]. However, much is still to be learnt in this field because tumours in those patients without such resistance mutations become resistant to chemotherapy, suggesting other unknown mechanisms are involved. 

## 2. Genetic Landscape of Human Gliomas 

Recent genome-wide sequencing studies of GBMs have provided insight into common genetic drivers of this tumour and have highlighted genetic differences between primary and secondary GBMs. Primary GBMs usually have one or more mutations in three main molecular pathways: Ras/RTK (receptor tyrosine kinase) pathway, p53 pathway, and the retinoblastoma (Rb) pathway [15,16]. Within the Ras/RTK pathway, epidermal growth factor receptor *(EGFR,* 30–50% of tumours) and phosphatase and tensin homolog (*PTEN,* 30%) are the most commonly mutated in GBM, although mutations in *NF1* and *RAS* have also been documented. Mutations within this pathway tend to enhance cellular proliferation. Of the p53 pathway, *TP53* (25%) itself is most commonly mutated in GBM. The *TP53* gene is normally activated following DNA damage to cells, inducing transcription of genes whose ultimate effects include apoptosis. Mutations in *TP53* are thought to have effects such as inhibition of apoptosis, stimulation of cell proliferation and neovascularisation, which are hallmarks of cancer [17]. Although a mutation in the Rb pathway is present in most GBMs, the *RB* gene itself is infrequently mutated, and instead, mutations in cyclin-dependent kinase inhibitor 2A *(CDKN2A*, 50% of tumours) are particularly common. *CDKN2A* is the locus for two tumour suppressor genes—*INK4A* and *P19-ARF*. In vitro and in vivo models have validated a number of such mutations as driving tumour growth and invasion. Mouse models have been particularly helpful in demonstrating how mutations in multiple pathways can co-operate together to accelerate tumourigenesis [18]. 

**Figure 1:**
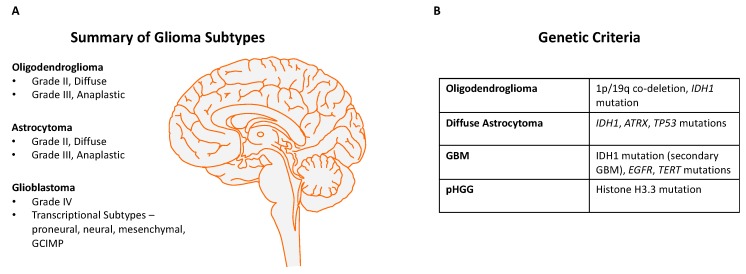
(**A**) Summary of glioma subtypes, including oligodendroglioma, astrocytoma and glioblastoma (GBM). The transcriptional subtypes of GBM are based on large-scale transcriptomics of human GBMs [25]. (**B**) The genetic features of these glioma subtypes, with the typical mutations seen in each category. The diagnosis of glioma is currently based on integration of classical histopathology with defining mutations (World Health Organisation (WHO) criteria). ‘pHGG’ refers to pediatric high-grade glioma, which has a different spectrum of mutations compared with adult GBMs. Illustration was prepared using the Motifolio drawing toolkit.

The *IDH1* (isocitrate dehydrogenase 1) mutation is characteristically found more commonly in secondary GBMs and also in LGGs [19], and although the mechanism by which this mutation contributes to carcinogenesis is still unclear, it is thought to act epigenetically through abnormal methylation of DNA [20]. The recent pathological classification of gliomas has been changed to take into account both classical histopathology and key genetic changes, such as *IDH1* mutant status, which predicts a better prognosis, and the presence or absence of 1p/19q co-deletions, *TERT* promoter mutations, and ATRX mutations [21,22]. A recent methylation classifier has further refined the molecular diagnosis of brain tumours [23]. Figure 1 summarises the integrated classification of diffuse gliomas based on the WHO classification 2016 [24]. 

## 3. Glioma Mouse Models 

The majority of glioma mouse models have employed cre/LoxP technology for specifically targeting cancer genes in certain neural tissues of interest. I will therefore describe this and related technologies before discussing examples of glioma mouse models in more detail. 

## 4. Cre/LoxP, Flp/FRT, RCAS Technology 

Site-specific recombination enables the creation of genetic alterations (for example, deletions, point mutations, duplications, and inversions). The first system to allow site-specific recombination in multicellular organisms was the flippase/flippase recognition target (Flp/FRT) system, and this was originally used in Drosophila [26]. Here, the flp recombinase mediates recombination between FRT sites in the genome. In the mouse, however, the commonest method for recombination is the use of the cre/LoxP system in which the cyclization recombinase (cre) mediates recombination between two LoxP sites. The LoxP sites are 34–base pair consensus sequences, each with a central 8-bp core spacer sequence determining the orientation of the LoxP site, and two inverted 13-bp flanking sequences that bind cre. The cre/LoxP system was initially implemented in mice in the early 1990s, and since then, it has been extensively used for creating conditional genetic alterations in vivo in many tissue types, including the brain [27]. Several hundred cre transgenic mouse lines have been developed in the last few decades for the study of organ or tissue-specific physiology and pathology [28], including development, cancer, aging, neurodegeneration, and other disease processes. 

Another system for producing targeted mutations in a tissue-specific manner is the RCAS (replication-competent ASLV long terminal repeat with a splice acceptor) vector system. Such vectors originate from the Rous sarcoma virus, which belongs to the avian sarcoma-leukosis virus (ASLV) family, and these vectors contain the src (oncogene) splice site and express an inserted gene (such as an oncogene) via a spliced message. Limitations of the RCAS system include the small size of the insert (2.5 kb), and the modest number of cells typically infected and that express the transgene [29], and for these reasons the cre/LoxP method is now more commonly employed for glioma and other cancer GEMMs. 

## 5. Cre Lines Used for Glioma GEMMs 

A number of cre lines have been utilised for the generation of glioma GEMMs. One such line is *nestin*-cre. Nestin is an intermediate filament protein expressed in neural stem cells and neural progenitors. Mice carrying the *nestin*-cre allele express cre from embryonic day 13, at which stage the embryonic neural progenitors are able to undergo differentiation into many cell types including astrocytes, neurons and oligodendrocytes. Therefore, in postnatal and adult mice carrying *nestin*-cre, cre is expressed throughout most of the central nervous system, eye, and also the kidneys—this was demonstrated by Dubois and colleagues who showed virtually complete cre-mediated recombination in these tissues by embryonic day 15.5 using LacZ based reporters [30,31]. However, the *nestin*-cre allele is insufficient for driving recombination in early embryonic ventricular zone neural progenitors and neural stem cells (before embryonic day 17.5), as determined using multiple cre-dependent reporters including LacZ [32]. An alternative cre line that is commonly used in glioma GEMMs is *hGFAP*-cre (driven by the human glial fibrillary acidic protein promoter), which is also expressed from pre-natal stages and in the majority of cell types in the brain and spinal cord [33]. In order to induce recombination in more specific groups of brain cells, alternative cre lines can be used. For example, *Olig2*-cre allows site-specific recombination in all oligodendrocyte lineages, *Syn1*-cre gives recombination specifically in neurons, and *Glast*-cre is a newer alternative line for recombination in neural stem cells in the subventricular zone (SVZ). 

Inducible cre lines are invaluable for studies where the timing of recombination needs to be controlled, requiring tamoxifen injections to induce cre expression. For example, *nestin*-creERT2 (cre fused to a mutant oestrogen ligand binding domain) and *GFAP*-creERT2 are inducible cre lines used for this purpose. These are particularly helpful for studying tumour origins from adult brain cells as opposed to embryonic cells (or other stage-specific neural precursors). The cre expression onset is controlled by specifying the age of the mice at which tamoxifen is administered. However, an important drawback of all of these cre lines is the specificity of the regions and cell types in which recombination occurs, in that there is typically recombination in other cells than those of interest. 

## 6. Glioma Mouse Models and Genetic Drivers 

One of the earliest oncogenes to be discovered in gliomas is the epidermal growth factor receptor (*EGFR*) gene [34], which is mutated and/or amplified in 50–60% of primary GBMs. EGFR is a cell-surface receptor that binds epidermal growth factor as its ligand and then signals via intracellular cascades, including the Ras-MAPK (mitogen-activated protein kinase) and phosphatidylinositol-3 kinase (PI3K)- protein kinase B (Akt) pathways. In primary GBMs, the variant III mutation of *EGFR* is particularly common, which is a deletion of exons 2–7 of the gene (the extracellular ligand binding domain) that promotes constitutive signalling from the resulting receptor. An early study, aimed at determining whether excessive EGFR signalling can induce gliomas in vivo, employed the RCAS vector system to introduce an *Egfr* activating mutation (the *EgfrvIII* deletion and second deletion that removes the intracellular regulatory kinase domain) in mice expressing the avian tumour virus receptor A (TVA) under control of brain cell specific promoters (nestin and glial fibrillary acidic protein (GFAP)). The vector was introduced into the frontal lobes and hippocampus. After 15 weeks, none of the mice developed gliomas. In contrast, when an *Egfr* activating mutation was introduced in the presence of *Cdkn2a* loss, gliomas arose at a high frequency particularly on the *nestin*-TVA (Ntv) background. The authors concluded that *Egfr* activating mutations alone are insufficient to generate gliomas but can cooperate with predisposing mutations such as those of *Cdkn2a* to produce these tumours [35]. Given the incidence of tumours was higher in Ntv compared with glial-specific GFAP-TVA (Gtv) mice, the authors suggested that the presence of these driving mutations in a neural stem cell lineage is a likely origin for gliomas. 

Another early glioma mouse model utilising the RCAS vector system was that by Holland and colleagues in 2000 [19], wherein a *Kras^G12D^* mutation and a constitutively active *Akt* mutant were virally transferred into the brain of mice using RCAS vectors. Each of these genes was insufficient to induce gliomas when expressed on their own. However, lesions histologically resembling human GBMs were observed when these mutations were expressed in combination with one other. Although it was initially believed that neither *Kras* or *Akt* mutations are present in human GBMs, more recent large-scale sequencing efforts have revealed *Kras* mutations of these cancers at a low frequency [36]. Moreover, the authors demonstrated elevated Ras pathway activation and increased Akt protein phosphorylation in the majority of GBMs, implying that upstream mutations are the likely cause of activation of these pathways that drive cellular proliferation and tumour growth. 

The tumour suppressor genes *TP53* and *PTEN* are mutated in ~30% and 40% of human GBMs, respectively, making these amongst the most frequently altered genes in this disease. Zheng et al. hypothesised that loss-of-function mutations in these two genes cooperate in driving gliomagenesis [37]. To explore this, they crossed a *hGFAP*-cre mouse with *Trp53* mutant and *Pten* knockout mice. The resulting mice developed grade III and grade IV gliomas at a median latency of approximately seven months [37]. Gliomaspheres with neural stem cell-like properties could be generated from these tumours, and the authors demonstrated that activation of myc was crucial in driving tumourigenesis in this model. Importantly, although *TP53* and *PTEN* mutations are commonly found in human low-grade gliomas as well as GBMs, all of the tumours generated in these mice were either grade III or IV. These findings were supported by data showing cooperation of *Trp53* and *Pten* with *Nf1* in another glioma GEMM derived from adult neural stem cells [38]. 

Zhu and colleagues explored the cooperation between the *EGFRvIII* mutation and other genes in gliomagenesis by also using transgenic mice [39]. They generated both an *EGFRvIII* transgenic mouse, in which this mutation was overexpressed at the *Col1a* locus, and an *EGFR* wild-type transgenic mouse with the human EGFR gene sequence inserted and over-expressed at the *Col1a* locus. These conditional transgenic mice required injections of cre into the brain for expression of the transgenes (cre was injected into the striatum of adult mice). Neither of these mutations was sufficient to initiate gliomas alone, but when each was expressed in combination with homozygous loss of *Pten* and *Ink4a*, both mutations were able to produce high-grade gliomas with a short latency. However, the *EGFRwt* allele produced tumours with a lower incidence and longer latency in comparison with a single *EGFRvIII* allele. Homozygous *EGFRvIII* was more tumorigenic than heterozygous *EGFRvIII*, although the difference was small. 

Given that *IDH1* is frequently mutated in human LGGs, often manifesting as the *IDH1^R132H^* mutation, it would be expected that these represent driver mutations. Conditional transgenic mice with *Idh1^R132H^* expression in the central nervous system were not found to generate gliomas (but perinatal brain haemorrhages instead) in earlier studies, although there was clear evidence that the same allele induces acute myeloid leukaemia in conditional transgenic mice when expressed in myeloid cells [40,41,42]. More recently, Bardella and colleagues generated an *Idh1^R132H^* knock-in mouse. Crossing this with *nestin*-cre mice led to pups with perinatal brain haemorrhages, as shown previously. However, when *Idh1^R132H^* mice were crossed with *nestin-creER^T2^* (an inducible cre) and tamoxifen administered at 5–6 weeks of age, these mice displayed increased neural stem and progenitor proliferation in the SVZ, as measured through increased GFAP, Ki67, and BrdU-labelled cells. At later stages, this expansion of SVZ neural progenitors led to microscopically detectable nodules, which were believed to represent possible glioma precursors [42]. This study highlights the importance of using stage-specific cellular recombination, given that an *Idh1* mutation led to early gliomagenesis in the adult SVZ but could not be detected from perinatal neural precursor recombination. It also adds weight to the argument that an *IDH1* mutation can be an initiating event in gliomagenesis, explaining the high frequency of clonal *IDH1* mutations in human gliomas and their presence in both primary and recurrent gliomas [43]. Table 1 summarises the key mouse models of gliomas.

## 7. Glioma Cell of Origin 

A well-studied but still controversial topic in glioma biology is which cell type gives rise to the tumour. Technological developments in glioma GEMMs have enabled important insights to be gleaned regarding the cell-of-origin of gliomas. Although it remains unclear which is the key cell type of origin, evidence suggests that the combination of genetic alterations has a major influence on whether one particular cell type can give rise to a glioma. Jacques and colleagues introduced combinations of *Trp53*/*Pten* and *Rb* mutations in adult subventricular zone (SVZ) neural stem cells (NSCs) and in astrocytes of mice by injections of adenovirus expressing cre (with and without the control of the GFAP promoter) [44]. Only SVZ stem cells produced tumours, whereas introducing these mutations into cultured astrocytes did not. Moreover, *Trp53* and *Pten* loss-of-function mutations together induced gliomas, whereas deletion of *Rb* in addition to *Trp53*/*Pten* loss led to primitive neuroectodermal tumours (PNETs) rather than gliomas. Importantly, despite containing the same mutations as those induced in the SVZ, mature astrocytes were unable to form tumours. 

A related study into the cellular origin of gliomas investigated the role of *Egfr* (activation) and *Cdkn2a* (loss) mutations in various brain cell types [45]. These mutations were introduced into in vitro cultured mouse astrocytes and neural stem cells. These mutated cells were subsequently transplanted into the brain (striatum) of severe combined immunodeficient (SCID) mice. When these mutations were introduced independently of each other, the cells did not transform into gliomas. With these mutations in combination however, gliomas were observed to arise from both astrocytes and neural stem cells. These results suggest that this particular combination of mutations rather than the cell type was more important in driving tumour formation. In particular, *Cdkn2a* loss led to the dedifferentiation of the mature astrocytes, enabling these cells to later be transformed if an activating *Egfr* mutation was introduced. The authors concluded that loss of *Cdkn2a* was a critical initial step in gliomagenesis that must precede *Egfr* activation if the latter is to trigger glioma formation, at least from mature astrocytes. 

To expand on these observations, Friedmann-Morvinski and colleagues performed direct lentiviral vector injections to cause p53/Nf1 knockdown or H-ras overexpression with p53 knockdown in neurons, astrocytes and neural stem cells of mice. Their models used cre-inducible lentiviral vectors, and *Syn1-cre* and *CamK2a-cre* transgenic mouse lines enabled recombination specifically in neurons. They found that all of these cell types generated malignant gliomas in mice with these genetic alterations and concluded that the mature cell types undergo dedifferentiation in response to defined oncogenic mutations to neural stem cells or progenitors, enabling tumour initiation and maintenance [46]. Although this elegantly demonstrated differentiated cell types can give rise to GBMs in vivo, this does not necessarily establish which cell type is the most likely origin. 

Another group investigated whether a particular cell type in the SVZ was particularly responsive to epidermal growth factor (EGF); they demonstrated in mice that infusion of EGF into the lateral ventricles caused increased proliferation of C-cells (transit amplifying progenitor cells that express nestin) in the SVZ, and these cells then invaded the brain parenchyma. Although no tumours occurred in this model, the study demonstrated that exogenous EGF can increase proliferation of neural stem cells through the wild-type *Egfr* activation [47]. It is unclear from this study alone though what the effect of the *EGFRvIII* mutation in absence of exogenous EGF would be on these cells. 

A further study elegantly used mosaic analysis with double markers (MADM) in mice with *p53*/*Nf1* inactivation in NSCs. MADM is a genetic mosaic system that uses cre/Loxp recombination to generate homozygous mutations in a small population of cells [48], labelling the mutant cells with green-fluorescent protein (GFP) and the wild-type cells with red-fluorescent protein (RFP). The authors used *hGFAP*-cre and *nestin*-cre to label neural stem cells and all lineages derived from them. Prior to GBM establishment, MADM-based lineage tracing identified aberrant growth only in oligodendrocyte precursor cells (OPCs), but not in NSCs or other NSC-lineages. Moreover, induction of *p53*/*Nf1* mutations directly in OPCs caused glioma formation, leading the authors to conclude that OPCs are the origin of glioma in this model, even if the initiating mutations occur in NSCs [49]. 

**Figure 2:**
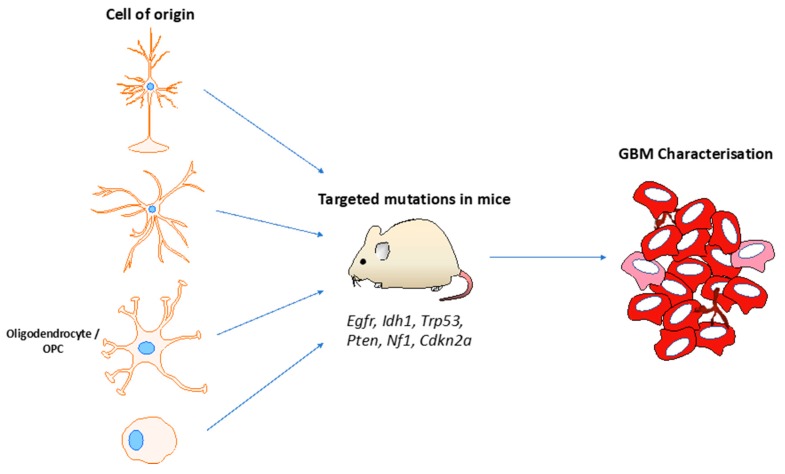
The use of genetically engineered mouse models for investigating the cell of origin of glioblastomas. The appropriate Cre transgenic mouse line can be selected for investigating the role of neurons, astrocytes, oligodendrocytes, oligodendrocyte precursor cells (OPC) or neural stem cells. Conditional mice carrying mutations in the relevant tumor suppressor genes or oncogenes are crossed with the cre line to determine the effect of these mutations in transforming the cell of interest. The glioblastomas (GBMs) generated are subject to phenotyping (such as transcriptional and histopathological) to determine their relevance to the human disease. Such models are also invaluable for exploring the cooperativity between genes of interest in gliomagenesis.

To test whether adult committed neural progenitors and adult OPCs can give rise to gliomas, a well-designed study leveraged the *Ascl1-creER^TM^* transgenic mouse line to target *Nf1*, *Trp53* and *Pten* loss-of -function mutations specifically in these cells to the exclusion of neural stem cells [50]. When mutations in all three genes were present, infiltrative GBMs with gene expression profiles similar to astrocytes were produced (type 1 tumours), whereas in the context of *Nf1−/−; Trp53−/−* these type 1 tumours were seen in addition to more circumscribed GBMs with expression profiles reflecting OPCs were observed (type 2 tumours). To confirm therefore that type 2 tumours are derived from adult OPCs, the investigators leveraged the *NG2-creER^TM^* transgenic line to express the same mutations specifically in adult OPCs only. These mice developed type 2 GBMs only. These data together suggest that the cell-of-origin influences the phenotype of GBMs, with adult OPCs and adult neural progenitors being capable of malignant transformation with appropriate mutations [51]. Figure 2 summarises the utility of GEMMs for investigating the glioma cell-of-origin. 

Recent work using sequencing data from human patients lends support to the subventricular zone (SVZ) being the origin of at least some GBMs [52]—the investigators performed deep sequencing of triple matched tissues from *IDH*-wild type GBM patients, including normal SVZ, tumour tissue and normal cerebral cortex. They found that normal SVZ displayed low level driver mutations (1% of tumour mutational burden) that were also present in the GBMs of the same patients in 56.3% of cases. The introduction of driver mutations in astrocyte-like NSCs in the SVZ in mice led to migration of these cells and formation of GBMs at distant brain regions. These data support cells in the SVZ as being potential origins of GBMs, although the precise cell type is unclear from this study alone, given that multiple neural progenitor populations reside in the SVZ. 

## 8. Sleeping Beauty Transposon-Based GEMMs 

A powerful platform for functional cancer gene discovery and also transgene insertion is the *Sleeping Beauty* (SB) transposon system. SB transposons are derived from the Tc1-Mariner family and are naturally found in salmonid fish. This transposon was reconstructed from phylogenetic data and was given the name ‘Sleeping Beauty’ to reflect that fact that it was ‘awakened from a long evolutionary sleep’. Ivics and colleagues discovered the sequence of the ancestral transposon from this class of fish, demonstrated to be two 250 bp terminal DNA sequences containing inverted repeats that flank an open reading frame that codes for the transposase enzyme [53]. *Sleeping Beauty* is therefore a two-component system composed of the transposon vector and the transposase enzyme. When these are present in the same cell, the transposase recognises the inverted repeats of the transposon and excises it from the donor locus. The transposon can then insert itself at a TA dinucleotide region elsewhere in the genome. In this way, the transposase catalyses a ‘cut and paste’ reaction of the transposon. Modifications to the inverted repeats have been made in order to improve transposition efficiency [54], and site-directed mutagenesis of the SB transposase produced alternative versions of the enzyme with different transposition efficiencies [55,56]. The first SB transposase was SB10, and modified versions were numerically labelled (SB11 and so on). 

Several SB insertional mutagenesis screens have been conducted in mice, successfully contributing to driver gene discovery for many cancers [57,58,59,60,61,62,63,64,65,66,67,68]. One constitutive SB screen in mice produced gliomas in the brain albeit with a low incidence [69,70]. The authors crossed these mice with a *P19Arf* allele, a known tumour suppressor gene in gliomas, which slightly increased the incidence of brain tumours. A mixture of anaplastic astrocytomas (grade III) and glioblastomas (grade IV) were generated. Genetic sequencing of the resulting 21 gliomas from this screen yielded 887 common integration sites (CIS—sites in the genome that are significantly recurrently ‘hit’ by the transposon more frequently than the background rate of insertions) and identified *Csf1* as a recurrently hit gene. Immunohistochemistry in human tumours demonstrated overexpression of CSF1 in high-grade astrocytomas, providing some support for a role of this gene in malignant glioma formation in humans as well as mice. However, the CIS genes did not include a number of well-established glioma genes such as *Egfr*, *Pdgfr*, and *Tp53*, and there were only single insertions found in other important tumour-specific genes including *Pten* and *Akt*. This may be explained by certain insertion site preference biases of SB or that SB-induced gliomas represent only a subset of gliomas that is not driven by the major cancer drivers in most human gliomas. It is therefore crucial that transposon-driven gliomas are studied in other genetically predisposed backgrounds in order to gain a more complete understanding of the cancer drivers, ideally with an alternative transposon system such as *piggyBac* as well. 

To enable screens to be performed for tissue-specific cancers, a conditional SB transposase allele was also developed that is active in the presence of cre expression and has been used for screens of many cancer types in mice. One study which employed this conditional SB transposon system for investigating gliomas used a *nestin*-cre allele on a *Trp53*-mutant background to drive expression of the SB transposase in mouse neural stem cells, although these did not directly generate tumours in vivo. In vitro culturing of embryonic neural stem cells derived from the SVZ of these mice demonstrated that these cells can be immortalised by mobilisation of SB. Immortalisation occurred significantly more frequently in cell lines with both *Trp53^R172H^* and mobilising SB than those with *Trp53^R172H^* alone, but not in lines with neither *Trp53^R172H^* nor mobilising SB. When these immortalised cells were subcutaneously transplanted into SCID mice, they generated tumours with a latency of 2–4 months. Through genetic sequencing for the SB insertions, the authors identified 106 CIS genes in the immortalised cell lines and 114 in the tumours, of which 34 CIS genes were present in both cohorts. Comparing the CIS genes from the immortalised cells with those of the tumours in mice showed that a further round of transposon mobilisation for in vivo tumour establishment was needed in addition to the insertions present in immortalised cells alone [71]. The authors were therefore able to categorise SB insertions according to whether they drove cellular proliferation in vitro or in vivo tumour growth or both, as part of a two-step process of cancer evolution. Amongst the CIS in the immortalised cell lines were a few known glioma genes, including *Pten*, and amongst the tumour CIS were genes such as *Pdgfrb* and *Nf1*. The study also identified putative cancer genes that were not previously linked with gliomas, such as *Met* and *Klf3* which were amongst their top-ranking tumour CIS. The CIS genes clustered into biological pathways that are thought to underlie gliomagenesis, in particular the Ras-MAPK and PI3K-Akt pathways, confirming that these major pathways that are mutated in human tumours can promote neural stem cell immortalisation in vitro and subcutaneous glioma formation. 

In addition to being used for in vivo genetic screens, *Sleeping Beauty* transposons have been proven to be a useful tool for inserting and expressing transgenes in cells both in vitro and in vivo, with relevance for glioma GEMMs [72]. *ATRX* (encoding a histone chaperone protein) is recurrently co-mutated with *TP53* in human GBMs. Elegant mouse modelling efforts used an SB transposase vector in conjunction with an ATRX knockdown sequence (shATRX) in a plasmid with flanking sequences recognized by the SB transposase to generate mice with ATRX knockdown [73]. The findings were that ATRX loss accelerated GBM formation in the context of *Trp53* loss and *Nras* overexpression, confirming ATRX acts as a tumour suppressor in vivo by impairing non-homologous end joining. This has clinical implications, in that ATRX GBMs in mice were more sensitive to double-stranded DNA damaging treatment such as irradiation and doxorubicin. 

*PiggyBac* is now widely used as a transposable vector for the efficient integration of transgenes in cells of interest, given that it has a relatively large cargo capacity [74,75]. 90% of paediatric GBMs carry a K27M mutation in a H3 variant, which is a gain-of-function mutation that leads to inhibition of the Polycomb repressive complex 2 (PRC2) [76]. A recent study used in utero electroporation of *piggyBac* vectors to integrate a H3.3K27M transgene into neural precursor cells (lining the ventricles) in the hindbrain and cerebral cortex of mouse embryos. When this was done in combination with CRISPR-cas9 induced *Trp53* loss, mice developed gliomas in both hindbrain and cortex within 8 months suggesting that *H3.3K27M* mutation cooperates with *Trp53* loss in gliomagenesis when they are present in embryonic neural precursor cells but not in adult neural precursors (given that no tumours were observed when these alleles were expressed under control of nestin and GFAP promoters) [77]. Although constitutive and conditional *piggyBac* genome-wide screens for cancer genes have been performed for haematological and other solid cancers in mice [78,79], such screens are still eagerly awaited for gliomas. 

## 9. CRISPR-Cas9 Genetic Engineering and Screens in Mice 

Clustered regularly interspaced short palindromic repeats (CRISPRs) were identified in *E.coli* in 1987 and in other bacteria and archaea a decade later [80,81]. The phage origin of these repeats and the identification of genes with putative nucleases associated with these repeats (CRISPR-associated) *cas*-genes led to the hypothesis and subsequent demonstration that the CRISPR-Cas system had a role in microbial adaptive immunity [82]. This is achieved by directing the Cas-nuclease to the incoming phage DNA by a guide RNA transcribed from the clustered repeats [83]. 

In contrast to the zinc-finger nuclease (ZFN) and transcription activator-like effector nuclease (TALEN) systems in which specificity is achieved by complex protein-nucleic acid interactions, the Cas-nuclease is directed to a genetic target by nucleic acid base pairing determined by a unique 20 nucleotide region of short guide RNA (sgRNA). Experimentally this sgRNA sequence can be adjusted to guide the nuclease to virtually any site in a complex genome [84]. The efficiency of the Cas-nuclease coupled with the simplicity with which it can be directed has resulted in its rapid adoption. The CRISPR-Cas9 system has been shown to be effective for manipulating genes in a variety of cell types from different organisms. When used as a nuclease, cleaved DNA is re-joined by an error-prone end-joining process resulting in small insertions and deletions (‘indels’) at the target site and concomitant loss of the gene’s function. Larger genetic alterations such as deletions and inversions can also be generated. In other applications the break generated by the nuclease will catalyse a process of homology directed repair if a suitable vector is also provided, resulting in replacement of one sequence (for instance, a defective copy) with a normal one provided by the vector, so called gene-editing. Studies have provided a cautionary note of potential off-target effects (unintended modifications at other sites in the genome) with this platform [85]. 

**Figure 3:**
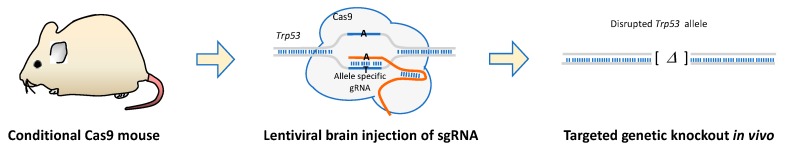
Somatic mutagenesis using clustered regularly interspaced short palindromic repeats (CRISPR)-cas9 for developing glioma genetically-engineered mouse models (GEMMs). A new and efficient method for generating somatic gene knockout is by crossing a conditional-Cas9 transgenic mouse with the appropriate cre for recombination in the cells of interest. Lentiviral delivery of single guide RNAs (sgRNAs) through stereotaxic brain injections will lead to knockout of the gene of interest (eg *Trp53*) only in the cells expressing Cas9 where cre-mediated recombination has occurred.

Glioma GEMMs based on CRISPR-Cas9 have demonstrated the versatility and efficiency of this technology for in vivo tumour models [86]. For example, in utero electroporation of the developing prosencephalon of multiple plasmids encoding Cas9 together with sgRNAs targeting *Nf1*, *Pten*, and *Trp53* led to the confirmed loss of these genes, and subsequent development of tumours histologically resembling human GBMs [87]. A modification of this is to use a conditional-Cas9 transgenic mouse line with a cre line to express cre in the cells of interest, and then use lentiviral injections to deliver and express sgRNAs in this cell population [88], as illustrated in Figure 3. This platform can be extended to validating putative tumour suppressor genes identified in human cancer genome sequencing studies. 

Over the last few years, a number of studies have demonstrated the usefulness of CRISPR-Cas9 in both positive and negative selection genome-wide screens, including in human cancer cells [89,90,91,92,93]. Such screens employ large lentiviral libraries with multiple sgRNAs per gene, and consequently require large starting populations of cells. A recent loss-of-function CRISPR screen conducted in conditional-Cas9 immune-competent mice used stereotaxic brain injections of a CRISPR library of sgRNAs targeting genes mutated in human cancers [94]. The adeno-associated virus (AAV) was used as a vector mediating integration of the sgRNA library. With this system, tumours that histologically resemble human GBMs were generated, and deep-sequencing revealed co-occurring mutations such as *B2m*-*Nf1* and *Mll3*-*Nf1* as putative driver combinations. These data demonstrate the utility of CRISPR-Cas9 for in vivo glioma functional tumour suppressor screens. Challenges with this approach however include the difficulty in achieving genome-wide coverage given the large number of cells that need to be transduced in order to cover a genome-wide library with multiple sgRNAs. 

## 10. Conclusions and Future Directions 

Mouse models have yielded landmark insights into the biology of gliomas in recent decades, and they continue to be indispensable for preclinical therapeutic testing. In order to maximise their relevance to human disease, glioma GEMMs need to faithfully recapitulate the molecular processes taking place, including genetic, epigenetic, transcriptional, and microenvironmental ones, amongst others. As technologies are being refined for engineering mutations found in human gliomas into the precise cell types of interest in the mouse brain, including by cre/LoxP recombination and viral- or transposon-based integration, our tools for investigating the mechanisms and origins of gliomagenesis are reaching new heights. 

Future efforts must focus on exploring how combinations of mutations seen in human tumours cooperate in driving glioma formation using these models. The generation and detailed characterisation of novel cre transgenic mouse lines will enable further targeting of mutations to increasingly specific brain cell types to investigate the glioma cell-of-origin. Oncogenic fusion events in GBM are beginning to receive increased appreciation as potential driver events with the advent of deeper genetic sequencing technologies, and appropriate genome engineering technologies are needed to validate these and explore their mechanisms with glioma GEMMs. Indeed, recent work has shown the potential of combining the RCAS-TVA system with CRISPR-Cas9 for somatic genome engineering of an oncogenic chromosomal translocation (such as *Myb*-*Qk*) in mice [95]. Perhaps most promising is the potential for genome-wide oncogene and tumour suppressor gene screens in vivo from *piggyBac* transposons and different versions of Cas9. Excitingly, these promise to provide large functional datasets in the investigation of GBM driver events and also potentially of resistance to novel targeted and immune therapies that will inform the next generation of clinical therapeutics. 

## Figures and Tables

**Table 1:** A summary of the key published mouse models of gliomas.

Cancer gene(s)	Technology	Latency	Pathology	Reference
*Egfr* activation (including *vIII*), *Cdkn2a*	RCAS, TVA	2 months for tumors in 13/25 mice	Low grade glioma	[35]
*Kras^G12D^*, *Akt*	RCAS	9 weeks for tumors in 7/27 mice	GBM	[19]
*Trp53* and *Pten* loss	hGFAP-cre (diverse glial cell types)	30 weeks, median latency	Grade III and Grade IV gliomas	[37]
*EGFRvIII, Ink4a,* and *Pten*	Cre injections in basal ganglia	7 weeks, median latency	GBM	[39]
*Idh1^R132H^*	Nestin-cre^ERT2^ (neural stem cells and progenitors)	6 weeks for precursors with 100% incidence	Glioma precursors	[42]
*Trp53, Pten and Rb* loss	Adenoviral-cre	8 months, mean latency	GBM (*Trp53* and *Pten*); PNET (*Trp53*, *Pten* and *Rb*)	[44]
*Nf1, Pten* and *Trp53* loss	hGFAP-cre (diverse glial cell types)	35 weeks, median latency	Grade III and Grade IV gliomas	[38]
*Trp53/Nf1* loss*;* or *H-ras* overexpression with *Trp53* loss	*SynI-cre* and *CamK2a-cre* (neurons)	6-10 weeks for SynI-cre, 9-12 months for *CamK2a-cre* (full penetrance)	GBM	[46]
*Nf1*, *Trp53* and *Pten* loss	*Ascl1-creER^TM^* (neural progenitors and OPCs).	40 weeks, median latency	GBM	[51]
*Atrx* loss*, Trp53* loss and *Nras* overexpression	*Sleeping beauty* transposon	50 days, median latency	GBM	[73]
*H3.3^K27M^, and Trp53* loss	*PiggyBac* transposons, CRISPR-cas9	9 months, full penetrance	GBM	[77]
*Nf1*, *Pten* and *Trp53* loss	CRISPR-cas9	14 weeks, complete penetrance	GBM	[87]
*PDGFB* expression; *Chk2*, *ATM* or *Trp53* loss	RCAS, TVA	60 days, median latency	GBM and low grade gliomas	[96]
*PDGFB* expression; *Trp53*, *Pten* or *Cdkn2a* loss	CRISPR-cas9	46-57 days, median latency	GBMs	[95]

RCAS = (Replication-Competent ASLV long terminal repeat (LTR) with a Splice acceptor). hGFAP = human glial fibrillary acidic protein. Nf1 = neurofibromatosis type 1. Chk2 = Checkpoint kinase 2. PDGFB = Platelet derived growth factor subunit B. Pten = phosphatase and tensin homolog. Cdkn2a = cyclin dependent kinase inhibitor 2A.

## References

[1] Hambardzumyan D., Gutmann D.H., Kettenmann H. (2016). The role of microglia and macrophages in glioma maintenance and progression. Nat. Neurosci..

[2] Chongsathidkiet P., Jackson C., Koyama S., Loebel F., Cui X., Farber S.H., Woroniecka K., Elsamadicy A.A., Dechant C.A., Kemeny H.R. (2018). Sequestration of T cells in bone marrow in the setting of glioblastoma and other intracranial tumors. Nat. Med..

[3] Noorani I., Petty G., Grundy P.L., Sharpe G., Willaime-Morawek S., Harris S., Thomas G.J., Nicoll J.A., Boche D. (2015). Novel association between microglia and stem cells in human gliomas: A contributor to tumour proliferation?. J. Pathol. Clin. Res..

[4] Hambardzumyan D., Parada L.F., Holland E.C., Charest A. (2011). Genetic modeling of gliomas in mice: New tools to tackle old problems. Glia.

[5] Lenting K., Verhaak R., Ter Laan M., Wesseling P., Leenders W. (2017). Glioma: Experimental models and reality. Acta Neuropathol..

[6] Kurzwelly D., Herrlinger U., Simon M. (2010). Seizures in patients with low-grade gliomas—Incidence, pathogenesis, surgical management, and pharmacotherapy. Adv. Tech. Stand. Neurosurg..

[7] Noorani I., Sanai N. (2017). Surgical Management of Incidental Gliomas. Neurosurg. Clin. N. Am..

[8] Mandonnet E., de Witt Hamer P., Pallud J., Bauchet L., Whittle I., Duffau H. (2014). Silent diffuse low-grade glioma: Toward screening and preventive treatment?. Cancer.

[9] Pallud J., Capelle L., Taillandier L., Badoual M., Duffau H., Mandonnet E. (2013). The silent phase of diffuse low-grade gliomas. Is it when we missed the action?. Acta Neurochir. (Wien.).

[10] Stupp R., Mason W.P., Van Den Bent M.J., Weller M., Fisher B., Taphoorn M.J., Belanger K., Brandes A.A., Marosi C., Bogdahn U. (2005). Radiotherapy plus concomitant and adjuvant temozolomide for glioblastoma. N. Engl. J. Med..

[11] Perry J.R., Laperriere N., O’Callaghan C.J., Brandes A.A., Menten J., Phillips C., Fay M., Nishikawa R., Cairncross J.G., Roa W. (2017). Short-Course Radiation plus Temozolomide in Elderly Patients with Glioblastoma. N. Engl. J. Med..

[12] Gilbert M.R., Dignam J.J., Armstrong T.S., Wefel J.S., Blumenthal D.T., Vogelbaum M.A., Colman H., Chakravarti A., Pugh S., Won M. (2014). A randomized trial of bevacizumab for newly diagnosed glioblastoma. N. Engl. J. Med..

[13] Jackson M., Hassiotou F., Nowak A. (2015). Glioblastoma stem-like cells: At the root of tumor recurrence and a therapeutic target. Carcinogenesis.

[14] Lee S.Y. (2016). Temozolomide resistance in glioblastoma multiforme. Genes Dis..

[15] Brennan C.W., Verhaak R.G., McKenna A., Campos B., Noushmehr H., Salama S.R., Zheng S., Chakravarty D., Sanborn J.Z., Berman S.H. (2013). The somatic genomic landscape of glioblastoma. Cell.

[16] Cancer Genome Atlas Research Network (2008). Comprehensive genomic characterization defines human glioblastoma genes and core pathways. Nature.

[17] Hanahan D., Weinberg R.A. (2011). Hallmarks of cancer: The next generation. Cell.

[18] Holland E.C., Celestino J., Dai C., Schaefer L., Sawaya R.E., Fuller G.N. (2000). Combined activation of Ras and Akt in neural progenitors induces glioblastoma formation in mice. Nat. Genet..

[19] Parsons D.W., Jones S., Zhang X., Lin J.C., Leary R.J., Angenendt P., Mankoo P., Carter H., Siu I.M., Gallia G.L. (2008). An integrated genomic analysis of human glioblastoma multiforme. Science.

[20] Molenaar R.J., Maciejewski J.P., Wilmink J.W., van Noorden C.J.F. (2018). Wild-type and mutated IDH1/2 enzymes and therapy responses. Oncogene.

[21] Eckel-Passow J.E., Lachance D.H., Molinaro A.M., Walsh K.M., Decker P.A., Sicotte H., Pekmezci M., Rice T., Kosel M.L., Smirnov I.V. (2015). Glioma Groups Based on 1p/19q, IDH, and TERT Promoter Mutations in Tumors. N. Engl. J. Med..

[22] Jaunmuktane Z., Capper D., Jones D.T., Schrimpf D., Sill M., Dutt M., Suraweera N., Pfister S.M., von Deimling A., Brandner S. (2019). Methylation array profiling of adult brain tumours: Diagnostic outcomes in a large, single centre. Acta Neuropathol. Commun..

[23] Capper D., Jones D.T., Sill M., Hovestadt V., Schrimpf D., Sturm D., Koelsche C., Sahm F., Chavez L., Reuss D.E. (2018). DNA methylation-based classification of central nervous system tumours. Nature.

[24] Laug D., Glasgow S.M., Deneen B. (2018). A glial blueprint for gliomagenesis. Nat. Rev. Neurosci..

[25] Verhaak R.G., Hoadley K.A., Purdom E., Wang V., Qi Y., Wilkerson M.D., Miller C.R., Ding L., Golub T., Mesirov J.P. (2010). Integrated genomic analysis identifies clinically relevant subtypes of glioblastoma characterized by abnormalities in PDGFRA, IDH1, EGFR, and NF1. Cancer Cell.

[26] Golic K.G., Lindquist S. (1989). The FLP recombinase of yeast catalyzes site-specific recombination in the Drosophila genome. Cell.

[27] Tsien J.Z. (2016). Cre-Lox Neurogenetics: 20 Years of Versatile Applications in Brain Research and Counting. Front. Genet..

[28] Friedel R.H., Wurst W., Wefers B., Kuhn R. (2011). Generating conditional knockout mice. Methods Mol. Biol..

[29] Fisher G.H., Orsulic S., Holland E., Hively W.P., Li Y., Lewis B.C., Williams B.O., Varmus H.E. (1999). Development of a flexible and specific gene delivery system for production of murine tumor models. Oncogene.

[30] Tronche F., Kellendonk C., Kretz O., Gass P., Anlag K., Orban P.C., Bock R., Klein R., Schütz G. (1999). Disruption of the glucocorticoid receptor gene in the nervous system results in reduced anxiety. Nat. Genet..

[31] Dubois N.C., Hofmann D., Kaloulis K., Bishop J.M., Trumpp A. (2006). Nestin-Cre transgenic mouse line Nes-Cre1 mediates highly efficient Cre/loxP mediated recombination in the nervous system, kidney, and somite-derived tissues. Genesis.

[32] Liang H., Hippenmeyer S., Ghashghaei H.T. (2012). A Nestin-cre transgenic mouse is insufficient for recombination in early embryonic neural progenitors. Biol. Open.

[33] Zhuo L., Theis M., Alvarez-Maya I., Brenner M., Willecke K., Messing A. (2001). hGFAP-cre transgenic mice for manipulation of glial and neuronal function in vivo. Genesis.

[34] Downward J., Yarden Y., Mayes E., Scrace G., Totty N., Stockwell P., Ullrich A., Schlessinger J., Waterfield M.D. (1984). Close similarity of epidermal growth factor receptor and v-erb-B oncogene protein sequences. Nature.

[35] Holland E.C., Hively W.P., DePinho R.A., Varmus H.E. (1998). A constitutively active epidermal growth factor receptor cooperates with disruption of G1 cell-cycle arrest pathways to induce glioma-like lesions in mice. Genes Dev..

[36] Ceccarelli M., Barthel F.P., Malta T.M., Sabedot T.S., Salama S.R., Murray B.A., Morozova O., Newton Y., Radenbaugh A., Pagnotta S.M. (2016). Molecular Profiling Reveals Biologically Discrete Subsets and Pathways of Progression in Diffuse Glioma. Cell.

[37] Zheng H., Ying H., Yan H., Kimmelman A.C., Hiller D.J., Chen A.J., Perry S.R., Tonon G., Chu G.C., Ding Z. (2008). p53 and Pten control neural and glioma stem/progenitor cell renewal and differentiation. Nature.

[38] Llaguno S.A., Chen J., Kwon C.H., Jackson E.L., Li Y., Burns D.K., Alvarez-Buylla A., Parada L.F. (2009). Malignant astrocytomas originate from neural stem/progenitor cells in a somatic tumor suppressor mouse model. Cancer Cell.

[39] Zhu H., Acquaviva J., Ramachandran P., Boskovitz A., Woolfenden S., Pfannl R., Bronson R.T., Chen J.W., Weissleder R., Housman D.E. (2009). Oncogenic EGFR signaling cooperates with loss of tumor suppressor gene functions in gliomagenesis. Proc. Natl. Acad. Sci. USA.

[40] Sasaki M., Knobbe C.B., Munger J.C., Lind E.F., Brenner D., Brüstle A., Harris I.S., Holmes R., Wakeham A., Haight J. (2012). IDH1(R132H) mutation increases murine haematopoietic progenitors and alters epigenetics. Nature.

[41] Sasaki M., Knobbe C.B., Itsumi M., Elia A.J., Harris I.S., Chio I.I., Cairns R.A., McCracken S., Wakeham A., Haight J. (2012). D-2-hydroxyglutarate produced by mutant IDH1 perturbs collagen maturation and basement membrane function. Genes Dev..

[42] Bardella C., Al-Dalahmah O., Krell D., Brazauskas P., Al-Qahtani K., Tomkova M., Adam J., Serres S., Lockstone H., Freeman-Mills L. (2016). Expression of Idh1(R132H) in the Murine Subventricular Zone Stem Cell Niche Recapitulates Features of Early Gliomagenesis. Cancer Cell.

[43] Lai A., Kharbanda S., Pope W.B., Tran A., Solis O.E., Peale F., Forrest W.F., Pujara K., Carrillo J.A., Pandita A. (2011). Evidence for sequenced molecular evolution of IDH1 mutant glioblastoma from a distinct cell of origin. J. Clin. Oncol..

[44] Jacques T.S., Swales A., Brzozowski M.J., Henriquez N.V., Linehan J.M., Mirzadeh Z., O’Malley C., Naumann H., Alvarez-Buylla A., Brandner S. (2010). Combinations of genetic mutations in the adult neural stem cell compartment determine brain tumour phenotypes. EMBO J..

[45] Bachoo R.M., Maher E.A., Ligon K.L., Sharpless N.E., Chan S.S., You M.J., Tang Y., DeFrances J., Stover E., Weissleder R. (2002). Epidermal growth factor receptor and Ink4a/Arf: Convergent mechanisms governing terminal differentiation and transformation along the neural stem cell to astrocyte axis. Cancer Cell.

[46] Friedmann-Morvinski D., Bushong E.A., Ke E., Soda Y., Marumoto T., Singer O., Ellisman M.H., Verma I.M. (2012). Dedifferentiation of neurons and astrocytes by oncogenes can induce gliomas in mice. Science.

[47] Doetsch F., Petreanu L., Caille I., Garcia-Verdugo J.M., Alvarez-Buylla A. (2002). EGF converts transit-amplifying neurogenic precursors in the adult brain into multipotent stem cells. Neuron.

[48] Zong H., Espinosa J.S., Su H.H., Muzumdar M.D., Luo L. (2005). Mosaic analysis with double markers in mice. Cell.

[49] Liu C., Sage J.C., Miller M.R., Verhaak R.G., Hippenmeyer S., Vogel H., Foreman O., Bronson R.T., Nishiyama A., Luo L. (2011). Mosaic analysis with double markers reveals tumor cell of origin in glioma. Cell.

[50] Kim E.J., Leung C.T., Reed R.R., Johnson J.E. (2007). In vivo analysis of Ascl1 defined progenitors reveals distinct developmental dynamics during adult neurogenesis and gliogenesis. J. Neurosci..

[51] Llaguno S.R., Wang Z., Sun D., Chen J., Xu J., Kim E., Hatanpaa K.J., Raisanen J.M., Burns D.K., Johnson J.E. (2015). Adult Lineage-Restricted CNS Progenitors Specify Distinct Glioblastoma Subtypes. Cancer Cell.

[52] Lee J.H., Lee J.E., Kahng J.Y., Kim S.H., Park J.S., Yoon S.J., Um J.Y., Kim W.K., Lee J.K., Park J. (2018). Human glioblastoma arises from subventricular zone cells with low-level driver mutations. Nature.

[53] Ivics Z., Hackett P.B., Plasterk R.H., Izsvak Z. (1997). Molecular reconstruction of Sleeping Beauty, a Tc1-like transposon from fish, and its transposition in human cells. Cell.

[54] Cui Z., Geurts A.M., Liu G., Kaufman C.D., Hackett P.B. (2002). Structure-function analysis of the inverted terminal repeats of the sleeping beauty transposon. J. Mol. Biol..

[55] Geurts A.M., Yang Y., Clark K.J., Liu G., Cui Z., Dupuy A.J., Bell J.B., Largaespada D.A., Hackett P.B. (2003). Gene transfer into genomes of human cells by the sleeping beauty transposon system. Mol. Ther..

[56] Mátés L., Chuah M.K., Belay E., Jerchow B., Manoj N., Acosta-Sanchez A., Grzela D.P., Schmitt A., Becker K., Matrai J. (2009). Molecular evolution of a novel hyperactive Sleeping Beauty transposase enables robust stable gene transfer in vertebrates. Nat. Genet..

[57] Takeda H., Wei Z., Koso H., Rust A.G., Yew C.C., Mann M.B., Ward J.M., Adams D.J., Copeland N.G., Jenkins N.A. (2015). Transposon mutagenesis identifies genes and evolutionary forces driving gastrointestinal tract tumor progression. Nat. Genet..

[58] Kas S.M., de Ruiter J.R., Schipper K., Annunziato S., Schut E., Klarenbeek S., Drenth A.P., van der Burg E., Klijn C., Ten Hoeve J.J. (2017). Insertional mutagenesis identifies drivers of a novel oncogenic pathway in invasive lobular breast carcinoma. Nat. Genet..

[59] Bard-Chapeau E.A., Nguyen A.T., Rust A.G., Sayadi A., Lee P., Chua B.Q., New L.S., De Jong J., Ward J.M., Chin C.K. (2014). Transposon mutagenesis identifies genes driving hepatocellular carcinoma in a chronic hepatitis B mouse model. Nat. Genet..

[60] Wu X., Northcott P.A., Dubuc A., Dupuy A.J., Shih D.J., Witt H., Croul S., Bouffet E., Fults D.W., Eberhart C.G. (2012). Clonal selection drives genetic divergence of metastatic medulloblastoma. Nature.

[61] Rahrmann E.P., Watson A.L., Keng V.W., Choi K., Moriarity B.S., Beckmann D.A., Wolf N.K., Sarver A., Collins M.H., Moertel C.L. (2013). Forward genetic screen for malignant peripheral nerve sheath tumor formation identifies new genes and pathways driving tumorigenesis. Nat. Genet..

[62] Moriarity B.S., Otto G.M., Rahrmann E.P., Rathe S.K., Wolf N.K., Weg M.T., Manlove L.A., LaRue R.S., Temiz N.A., Molyneux S.D. (2015). A Sleeping Beauty forward genetic screen identifies new genes and pathways driving osteosarcoma development and metastasis. Nat. Genet..

[63] Chen L., Jenjaroenpun P., Pillai A.M., Ivshina A.V., Ow G.S., Efthimios M., Zhiqun T., Tan T.Z., Lee S.C., Rogers K. (2017). Transposon insertional mutagenesis in mice identifies human breast cancer susceptibility genes and signatures for stratification. Proc. Natl. Acad. Sci. USA.

[64] Mann M.B., Black M.A., Jones D.J., Ward J.M., Yew C.C., Newberg J.Y., Dupuy A.J., Rust A.G., Bosenberg M.W., McMahon M. (2015). Transposon mutagenesis identifies genetic drivers of Braf(V600E) melanoma. Nat. Genet..

[65] Dupuy A.J., Akagi K., Largaespada D.A., Copeland N.G., Jenkins N.A. (2005). Mammalian mutagenesis using a highly mobile somatic Sleeping Beauty transposon system. Nature.

[66] Mann K.M., Ward J.M., Yew C.C., Kovochich A., Dawson D.W., Black M.A., Brett B.T., Sheetz T.E., Dupuy A.J., Chang D.K. (2012). Sleeping Beauty mutagenesis reveals cooperating mutations and pathways in pancreatic adenocarcinoma. Proc. Natl. Acad. Sci. USA.

[67] Rahrmann E.P., Collier L.S., Knutson T.P., Doyal M.E., Kuslak S.L., Green L.E., Malinowski R.L., Roethe L., Akagi K., Waknitz M. (2009). Identification of PDE4D as a proliferation promoting factor in prostate cancer using a Sleeping Beauty transposon-based somatic mutagenesis screen. Cancer Res..

[68] De La Rosa J., Weber J., Friedrich M.J., Li Y., Rad L., Ponstingl H., Liang Q., De Quirós S.B., Noorani I., Metzakopian E. (2017). A single-copy Sleeping Beauty transposon mutagenesis screen identifies new PTEN-cooperating tumor suppressor genes. Nat. Genet..

[69] Bender A.M., Collier L.S., Rodriguez F.J., Tieu C., Larson J.D., Halder C., Mahlum E., Kollmeyer T.M., Akagi K., Sarkar G. (2010). Sleeping beauty-mediated somatic mutagenesis implicates CSF1 in the formation of high-grade astrocytomas. Cancer Res..

[70] Collier L.S., Adams D.J., Hackett C.S., Bendzick L.E., Akagi K., Davies M.N., Diers M.D., Rodriguez F.J., Bender A.M., Tieu C. (2009). Whole-body sleeping beauty mutagenesis can cause penetrant leukemia/lymphoma and rare high-grade glioma without associated embryonic lethality. Cancer Res..

[71] Koso H., Takeda H., Yew C.C., Ward J.M., Nariai N., Ueno K., Nagasaki M., Watanabe S., Rust A.G., Adams D.J. (2012). Transposon mutagenesis identifies genes that transform neural stem cells into glioma-initiating cells. Proc. Natl. Acad. Sci. USA.

[72] Wiesner S.M., Decker S.A., Larson J.D., Ericson K., Forster C., Gallardo J.L., Long C., Demorest Z.L., Zamora E.A., Low W.C. (2009). De novo induction of genetically engineered brain tumors in mice using plasmid DNA. Cancer Res..

[73] Koschmann C., Calinescu A.A., Nunez F.J., Mackay A., Fazal-Salom J., Thomas D., Mendez F., Kamran N., Dzaman M., Mulpuri L. (2016). ATRX loss promotes tumor growth and impairs nonhomologous end joining DNA repair in glioma. Sci. Transl. Med..

[74] Wang W., Lin C., Lu D., Ning Z., Cox T., Melvin D., Wang X., Bradley A., Liu P. (2008). Chromosomal transposition of PiggyBac in mouse embryonic stem cells. Proc. Natl. Acad. Sci. USA.

[75] Liang Q., Kong J., Stalker J., Bradley A. (2009). Chromosomal mobilization and reintegration of Sleeping Beauty and PiggyBac transposons. Genesis.

[76] Bender S., Tang Y., Lindroth A.M., Hovestadt V., Jones D.T., Kool M., Zapatka M., Northcott P.A., Sturm D., Wang W. (2013). Reduced H3K27me3 and DNA hypomethylation are major drivers of gene expression in K27M mutant pediatric high-grade gliomas. Cancer Cell.

[77] Pathania M., De Jay N., Maestro N., Harutyunyan A.S., Nitarska J., Pahlavan P., Henderson S., Mikael L.G., Richard-Londt A., Zhang Y. (2017). H3.3(K27M) Cooperates with Trp53 Loss and PDGFRA Gain in Mouse Embryonic Neural Progenitor Cells to Induce Invasive High-Grade Gliomas. Cancer Cell.

[78] Rad R., Rad L., Wang W., Cadinanos J., Vassiliou G., Rice S., Campos L.S., Yusa K., Banerjee R., Li M.A. (2010). PiggyBac transposon mutagenesis: A tool for cancer gene discovery in mice. Science.

[79] Rad R., Rad L., Wang W., Strong A., Ponstingl H., Bronner I.F., Mayho M., Steiger K., Weber J., Hieber M. (2015). A conditional piggyBac transposition system for genetic screening in mice identifies oncogenic networks in pancreatic cancer. Nat. Genet..

[80] Ishino Y., Shinagawa H., Makino K., Amemura M., Nakata A. (1987). Nucleotide sequence of the iap gene, responsible for alkaline phosphatase isozyme conversion in Escherichia coli, and identification of the gene product. J. Bacteriol..

[81] Mojica F.J., Diez-Villasenor C., Soria E., Juez G. (2000). Biological significance of a family of regularly spaced repeats in the genomes of Archaea, Bacteria and mitochondria. Mol. Microbiol..

[82] Barrangou R., Fremaux C., Deveau H., Richards M., Boyaval P., Moineau S., Romero D.A., Horvath P. (2007). CRISPR provides acquired resistance against viruses in prokaryotes. Science.

[83] Jinek M., Chylinski K., Fonfara I., Hauer M., Doudna J.A., Charpentier E. (2012). A programmable dual-RNA-guided DNA endonuclease in adaptive bacterial immunity. Science.

[84] Cong L., Ran F.A., Cox D., Lin S., Barretto R., Habib N., Hsu P.D., Wu X., Jiang W., Marraffini L.A. (2013). Multiplex genome engineering using CRISPR/Cas systems. Science.

[85] Fu Y., Foden J.A., Khayter C., Maeder M.L., Reyon D., Joung J.K., Sander J.D. (2013). High-frequency off-target mutagenesis induced by CRISPR-Cas nucleases in human cells. Nat. Biotechnol..

[86] Mao X.Y., Dai J.X., Zhou H.H., Liu Z.Q., Jin W.L. (2016). Brain tumor modeling using the CRISPR/Cas9 system: State of the art and view to the future. Oncotarget.

[87] Zuckermann M., Hovestadt V., Knobbe-Thomsen C.B., Zapatka M., Northcott P.A., Schramm K., Belic J., Jones D.T., Tschida B., Moriarity B. (2015). Somatic CRISPR/Cas9-mediated tumour suppressor disruption enables versatile brain tumour modelling. Nat. Commun..

[88] Platt R.J., Chen S., Zhou Y., Yim M.J., Swiech L., Kempton H.R., Dahlman J.E., Parnas O., Eisenhaure T.M., Jovanovic M. (2014). CRISPR-Cas9 knockin mice for genome editing and cancer modeling. Cell.

[89] Wang T., Wei J.J., Sabatini D.M., Lander E.S. (2014). Genetic screens in human cells using the CRISPR-Cas9 system. Science.

[90] Shalem O., Sanjana N.E., Hartenian E., Shi X., Scott D.A., Mikkelsen T.S., Heckl D., Ebert B.L., Root D.E., Doench J.G. (2014). Genome-scale CRISPR-Cas9 knockout screening in human cells. Science.

[91] Koike-Yusa H., Li Y., Tan E.P., Velasco-Herrera Mdel C., Yusa K. (2014). Genome-wide recessive genetic screening in mammalian cells with a lentiviral CRISPR-guide RNA library. Nat. Biotechnol..

[92] Gilbert L.A., Horlbeck M.A., Adamson B., Villalta J.E., Chen Y., Whitehead E.H., Guimaraes C., Panning B., Ploegh H.L., Bassik M.C. (2014). Genome-Scale CRISPR-Mediated Control of Gene Repression and Activation. Cell.

[93] Konermann S., Brigham M.D., Trevino A.E., Joung J., Abudayyeh O.O., Barcena C., Hsu P.D., Habib N., Gootenberg J.S., Nishimasu H. (2015). Genome-scale transcriptional activation by an engineered CRISPR-Cas9 complex. Nature.

[94] Chow R.D., Guzman C.D., Wang G., Schmidt F., Youngblood M.W., Ye L., Errami Y., Dong M.B., Martinez M.A., Zhang S. (2017). AAV-mediated direct in vivo CRISPR screen identifies functional suppressors in glioblastoma. Nat. Neurosci..

[95] Oldrini B., Curiel-García Á., Marques C., Matia V., Uluçkan Ö., Graña-Castro O., Torres-Ruiz R., Rodriguez-Perales S., Huse J.T., Squatrito M. (2018). Somatic genome editing with the RCAS-TVA-CRISPR-Cas9 system for precision tumor modeling. Nat. Commun..

[96] Squatrito M., Brennan C.W., Helmy K., Huse J.T., Petrini J.H., Holland E.C. (2010). Loss of ATM/Chk2/p53 pathway components accelerates tumor development and contributes to radiation resistance in gliomas. Cancer Cell.

